# A New Strain of Virus Discovered in China Specific to the Parasitic Mite *Varroa destructor* Poses a Potential Threat to Honey Bees

**DOI:** 10.3390/v13040679

**Published:** 2021-04-15

**Authors:** Gongwen Chen, Shuai Wang, Shuo Jia, Ye Feng, Fuliang Hu, Yanping Chen, Huoqing Zheng

**Affiliations:** 1 College of Animal Sciences, Zhejiang University, Hangzhou 310058, China; chengongwen84@foxmail.com (G.C.); troywang0420@foxmail.com (S.W.); jiashuoupup@163.com (S.J.); flhu@zju.edu.cn (F.H.); 2 Insitutute for Translational Medicine, Zhejiang University School of Medicine, Hangzhou 310058, China; pandafengye@zju.edu.cn; 3 USDA-ARS Bee Research Laboratory, Beltsville, MD 20705, USA

**Keywords:** virus, *Varroa* mite, honey bees, genome, prevalence, replication

## Abstract

The ectoparasitic mite, *Varroa destructor*, feeds directly on honey bees and serves as a vector for transmitting viruses among them. The *Varroa* mite causes relatively little damage to its natural host, the Eastern honey bee (*Apis cerana*) but it is the most devastating pest for the Western honey bee (*Apis mellifera*). Using Illumina HiSeq sequencing technology, we conducted a metatranscriptome analysis of the microbial community associated with *Varroa* mites. This study led to the identification of a new Chinese strain of *Varroa destructor* virus-2 (VDV-2), which is a member of the *Iflaviridae* family and was previously reported to be specific to *Varroa* mites. A subsequent epidemiological investigation of Chinese strain of VDV-2 (VDV-2-China) showed that the virus was highly prevalent among *Varroa* populations and was not identified in any of the adult workers from both *A. mellifera* and *A.* *cerana* colonies distributed in six provinces in China, clearly indicating that VDV-2-China is predominantly a *Varroa*-adapted virus. While *A. mellifera* worker pupae exposed to less than two *Varroa* mites tested negative for VDV-2-China, VDV-2-China was detected in 12.5% of the *A. mellifera* worker pupae that were parasitized by more than 10 *Varroa* mites, bringing into play the possibility of a new scenario where VDV-2 could be transmitted to the honey bees during heavy *Varroa* infestations. Bioassay for the VDV-2-China infectivity showed that *A. cerana* was not a permissive host for VDV-2-China, yet *A. mellifera* could be a biological host that supports VDV-2-China’s replication. The different replication dynamics of the virus between the two host species reflect their variation in terms of susceptibility to the virus infection, posing a potential threat to the health of the Western honey bee. The information gained from this study contributes to the knowledge concerning genetic variabilities and evolutionary dynamics of Varroa-borne viruses, thereby enhancing our understanding of underlying molecular mechanisms governing honey bee Varroosis.

## 1. Introduction

The ectoparasitic mite, *Varroa destructor* (hereafter referred to as the *Varroa* mite) [1], is the world’s most detrimental pest of the Western honey bee, *Apis mellifera*, which is the primary managed pollinator in agricultural systems and natural ecosystems. Since its host expansion from the Eastern honey bee *Apis cerana* to *A. mellifera* in the mid-1900s, the *Varroa* mite has now spread to every region where honey bees are kept with the exception of Australia, and has been catastrophic for the beekeeping industry worldwide [2,3,4]. While *Varroa* mites cause limited damage to its original host *A. cerana*, they inflict profound damage on the *A. mellifera* hosts through the intake of their fat body tissue and hemolymph from immature brood and adult honey bees [5,6]. The repeated feeding results in a significant decline in the *A. mellifera* host’s vigor, immunity, weight, and lifespan, and leads to the eventual collapse of entire colonies [6,7,8,9,10,11,12,13]. *Varroa* mite infestations in *A. mellifera* colonies have been cited as strong predictive markers for honey bee colony mortality during winter [14,15,16,17,18]. The massive *Varroa* mite infestation causes a devastating honey bee disease called Varroosis and has destroyed millions of *A. mellifera* colonies since its establishment in various parts of the world [19].

The ability of the *Varroa* mite to carry and spread multiple debilitating viruses has added another layer to the complicated and dire situation of honey bee health [10,20,21]. Because mites feed and move regularly between brood and adult bees, they have the potential to act as vectors of bee viruses. The term Bee Parasitic Mite Syndrome has been used to describe a disease complex in which colonies are simultaneously infected with viruses and infested with *Varroa* mites [22]. Viral infections in honey bee colonies have often been reported to be involved in the collapse of bee colonies infested with *V. destructor*. Several bee viruses, including Acute bee paralysis virus (ABPV), Black queen cell virus (BQCV), Chronic bee paralysis virus (CBPV), Deformed wing virus (DWV), Kashmir bee virus (KBV), Sacbrood virus (SBV) and Israeli acute paralysis virus (IAPV), have been documented to be aggravated and transmitted by *Varroa* mites in field settings [23,24,25,26,27,28,29]. The field observations of the association between *Varroa* mite infestations and virus infections in honey bees led to important laboratory experiments, which resulted in the conclusion that *Varroa* mite is a highly efficient mechanical and/or biological vector of honey bee viruses, and it drives the spread of viruses in honey bee colonies [30,31,32,33]. Further studies showed that, in addition to facilitating the virus transmission, the *Varroa* mite activates bee viruses by suppressing the honey bees’ immune responses and triggering the replications of viruses in immunosuppressed hosts [11,30,33,34,35,36,37,38,39].

Of all honey bee viruses vectored by *Varroa* mites, DWV is the most common and widespread honeybee viruses and infects a wide range of host species [40,41]. The deadly association between DWV and *Varroa* mites is particularly worrisome [42]. The *Varroa* mite magnifies the negative effects of DWV infections and drives the selection of highly virulent DWV strains which has altered the viral landscape and resulted in the destruction of millions of bee colonies worldwide [19]. It has been documented that *Varroa* vectoring has resulted in the emergence of at least three master variants in honeybees: DWV-A, DWV-B (or Varroa destructor virus-1, VDV-1), and DWV-C [29,43,44]. Prior to the spread of *Varroa* invasion, DWV-A was a predominant master variant and bees infected with DWV-A exhibited low viral titers and were non-symptomatic [45]. However, *Varroa* vectoring caused genetic mutations among DWV populations, leading to a new master variant, Varroa destructor virus 1 (VDV1, also referred to as DWV-B) which shares 84.4% nucleotide sequence identify with DWV-A and is closely linked to overwinter honeybee worker loss [43,46,47,48]. Recently, a third master variant, DWV-C which is distinct from the aforementioned DWV-A and DWV-B, was reported [49] and, in combination with DWV-A, was linked to the death of overwintering colonies in which the *Varroa* mite infestation was not at economic injury levels [18,50]. Although DWV-A, DWV-B, and DWV-C belong to the same viral cloud of DWV species complex, their distribution, prevalence, and virulence vary significantly at colony level [51].

So far, more than 20 viruses that cause honey bee infections ranging from asymptomatic to severe, debilitating illness and contribute to elevated colony losses worldwide have been identified [10,20,52]. The majority of honey bee viruses are positive-sense single-stranded RNA viruses that fall into two families within the order Picornavirales. Viral families in the order Picornavirales share the following properties: (i) auto-proteolytically processed polyprotein(s), (ii) a common three-domain replication block (Hel-Pro-Pol domain consisting of a superfamily III helicase, a proteinase with a chymotrypsin-like structure, and a superfamily I RNA-dependent RNA polymerase), and (iii) capsid proteins organized in a module containing three related jelly-roll domains which form non-enveloped icosahedral virions approximately 30 nm in diameter with a pseudo-T = 3 symmetry [53]. Common bee viruses, including ABPV, BQCV, KBV, and IAPV are monopartite bicistronic with non-structural gene at the 5′ end and structural genes at the 3′ end of genomes and are classified in the Dicistroviridae family [54]. Other common bee viruses including DWV, SBV, and SBPV are monopartite monocistronic with structural genes at the 5′ end and non-structural genes at the 3′ end of genomes and are classified in the Iflaviridae family [55]. CBPV and Lake Sinai viruses (LSVs) are also common bee-infecting viruses but they have not been classified into taxa [44].

Additionally, recent studies have identified novel RNA viruses that are exclusively associated with *V. destructor* [56,57,58]. Varroa destructor virus-1 (VDV-1) is a member of the Iflaviridae family [55] and was the first isolated virus from *Varroa* mites [46]. VDV-1 actively infects honey bees and novel recombinants between DWV and VDV-1 were found to prevail in *Varroa* mite infested honeybee colonies [48,59,60]. The in silico analysis of the transcriptome data of *Varroa* mites collected in the U.S.A. and Europe led to the identification of RNA viruses including *Varroa destructor* virus 2 (VDV-2) and VDV-3, which were found to be able to replicate in the mite but not in the honey bee, suggesting that they are specific to *Varroa* mites [56,58].

In China, both *A. mellifera* and *A. cerana* are often kept in close proximity for honey production and crop pollination, which has created unique opportunities for the spread of *Varroa* mites and the transmission dynamics of *Varroa*-borne viruses between two host species. In the present study, we employed a high-throughput next-generation sequencing technology to detect the viruses associated with *Varroa* mites. Our study led to the identification of a new Chinese strain of VDV-2 (VDV-2-China). We conducted the subsequent studies to investigate the incidence and prevalence of VDV-2-China in the *Varroa* mites and honey bees, including *A. mellifera* and *A. cerana*, in different geographic regions of China. Furthermore, we investigated and compared the VDV-2-China replications in the two honey bee species under the laboratory conditions. The information gained from this study contributes to the existing body of literature concerning genetic variabilities and evolutionary dynamics of Varroa-borne viruses, thereby enhancing our understanding of underlying molecular mechanisms governing honey bee Varroosis and improving the management of *Varroa* mite infestation in honey bees.

## 2. Material and Methods

### 2.1. Ethics Statement

The Italian honey bee (*Apis mellifera ligustica*) and the Eastern honey bees (*Apis cerana cerana*) used in the study are neither endangered nor protected species. No specific permits were required. 

### 2.2. Sample Collection

For the RNA sequencing (RNA-Seq) analysis, honey bee combs were taken out from the *A. mellifera* colonies that are maintained at an apiary of the College of Animal Sciences, Zhejiang University, Hangzhou, China and adult female *V. destructor* were collected from the sealed drone brood cells of the combs. 

For the epidemiological study, *Varroa* samples were collected from *A. mellifera* colonies and *A. cerana* colonies in an apiary located in Zhejiang province. For *A. mellifera* colonies, the brood cells in the frames were opened and pupae were removed individually with a pair of tweezers to check for the presence of *Varroa* mites. Individual worker pupae with one *Varroa* mite parasitizing on them when the brood cell was opened were designated as ng low count of mites, while worker pupae with more than 10 *Varroa* mites, including the foundresses and the offspring, were defined as having high count of mites For *A. cerana* colonies, our sampling only focused on the adult workers as *Varroa* mites express strong avoidance to the *A. cerana* worker brood in the field and no *Varroa*-infested *A. cerana* worker brood was identified [61,62].

According to our field survey over the past few years, only *V. destructor* and *Varroa. underwoodi* were found in *A. cerana* colonies [62,63]. The two species are very different in morphology and *V. underwoodi* occurs at a much lower abundance, compared to *V. destructor* [63]. The *Varroa* mites collected from both *A. mellifera* and *A. cerana* colonies are confirmed to be *V. destructor* by morphological identification and molecular identification [63]. 

In addition, 30 adult workers were collected by brushing off bees from a brood frame from each of 180 *A. mellifera* colonies and 180 *A. cerana* colonies distributed in six provinces (Zhejiang, Gansu, Hubei, Jiangxi, Guangdong, and Yunnan) of China. These samples were placed individually in a microcentrifuge tube, snapped frozen in liquid nitrogen, and transferred to Zhejiang University for subsequent molecular analysis.

### 2.3. RNA Sequencing 

*Varroa* mites from three *A. mellifera* colonies were used for RNA-Seq analysis. For each colony, total RNA was extracted from the pool of five *V. destructor* mites according to the manufacturer’s instructions (Aidlab, Beijing, China). The concentration of RNA samples was measured by using a Nanodrop2000 and the integrity of the RNA was confirmed using the Agilent 2100 BioAnalyzer. The Ribo-ZeroTM kit (Epicentre, Beijing, China) was used to remove ribosomal RNA. cDNA was synthesized from the RNA using random hexamer primers and the QIAGEN Reverse Transcription Kit following the manufacturer’s instructions and submitted to a commercial company (Novogene Co., Beijing, China) for transcriptome sequencing analysis. The Illumina Hiseq sequencing platform was used for transcriptome sequencing. An Illumina PE library was constructed for 2 × 150 bp sequencing, and the obtained sequencing data were subjected to quality control. The Illumina reads were processed to remove low-quality sequences and adaptor by Trimmomatic v0.4 (PMID: 24695404).

The reads of RNA sequencing data were compared with sequences in the database through Bowtie2 with default parameters [64], and reads of *V. destructor* and *A. mellifera* were eliminated. Then, the remaining reads were assembled by Trinity software with default parameters [65]. The assembled contigs were compared to the sequences in Nucleotide Sequence Database (NT) and Non-Redundant Protein Sequence Database (NR) of National Center for Biotechnology Information (NCBI) by BLASTN [66] and BLASTX with E value threshold to be 1 × 10^−15^ [67]. Contigs were removed if they meet any of the following conditions: (1) contigs with a length of less than 500 bp; (2) contigs with a similarity of nucleic acid sequence to known viral sequences greater than 90%; and (3) contigs with a similarity of amino acid sequence to known viral protein sequences less than 25%. The raw sequencing data were submitted into the NCBI’s Sequence Read Archive (SRA) database with ID # SUB9162165.

### 2.4. Virus Discovery and Phylogenetic Analysis

A full viral genome sequence with high homology to VDV-2 was identified in the *V. destructor*’s transcriptome, which showed a sequencing depth > 150× in all of the three sequenced samples. The genomic organization and location of the conserved motifs for the structural and non-structural proteins were determined by a multiple sequence alignment of *iflaviruses*. The protein domains were defined by pairwise amino acid sequence alignment between VDV-2-China and VDV-2-UK (QFS19921) whose functional regions have been identified and annotated and further confirmed by Pfam matches. The viral genome sequence identified in this study was submitted to Genbank with an accession number (MW590582). 

To classify the newly discovered virus, the phylogenetic analysis was conducted in MEGA 7 [68] using the neighbor-joining method, which is based on a Poisson model [69], and the maximum likelihood method [70]. Taxa used for generating an unrooted tree based on amino acid sequences of the putative structural polyprotein included: Chinese strain of VDV-2 obtained in the present study; *Iflaviridae*-Deformed wing virus (DWV) (AY292384), DWV-C (SAMEA3249778), Ectropis obliqua virus (AY365064), Infectious flacherie virus (AB000906), Perina nuda virus (AF323747), Sacbrood virus (AF092924), Varroa destructor virus 1 (AY251269), Varroa destructor virus 2 (QFS19921), Brevicoryne brassicae picorna-like virus (EF517277), Slow bee paralysis virus (EU035616), and Slow bee paralysis virus (Harpenden) (GU938761); *Dicistroviridae*-Acute bee paralysis virus (AF150629), Cricket paralysis virus (AF218039), Drosophila C virus (AF014388), Kashmir bee virus (AY27571O), Rhopalosiphum padi virus (AF022937), Solenopsis invicta virus 1 (AY634314), Taura syndrome virus (AF277675), and Israeli acute paralysis virus (EF219380); *Secoviridae*-Parsnip yellow fleck virus (D14066), Cowpea severe mosaic virus (M83830), Maize chlorotic dwarf virus (NP619716), Bean pod mottle virus (NP612349, Radish mosaic virus (YP001911126), and Cowpea mosaic virus (NP613283); *Picornaviridae*-Poliovirus (VOl149), Encephalomyocarditis virus (M81861), Aimelvirus 1 strain (ASH99046), and Rabbit kobuvirus (YP009118268). A phylogenetic tree based on the amino acid sequence of RNA-dependent RNA polymerase (RdRp) was also generated to show the relationship between the VDV-2-China and other members of the family *Iflaviridae*.

Sequences were aligned individually using Multiple Sequence Comparison by Log-Expectation (MUSCLE) 3.8 [71] that was implemented in Geneious Prime 2019.0.4 [72]. The phylogenetic trees were assessed by bootstrap replication (N = 1000 replicates) and bootstrap values of 60% were regarded as providing evidence for the phylogenetic grouping.

### 2.5. RNA Extraction and Reverse Transcription Polymerase Chain Reaction (RT-PCR) 

Samples were individually crushed into fine powder in liquid nitrogen and homogenized in TRIzol Reagent for RNA extraction. A cDNA synthesis was performed using PrimeScript RT Master Mix (Takara, Beijing, China) with 200 ng of *V. destructor* RNA and 800 ng honey bee RNA. 

Polymerase chain reaction (PCR) amplifications were conducted using KOD FX (TOYOBO, Shanghai, China) with 1 μLof cDNA. The VDV-2-China specific primers were Forward: 5′-AACACCAGACGAACAAGGCT-3′; Reverse: 5′-TTGGCTATGGTGAAGGCTCG-3′. A fragment length of 285 bp of VDV-2-China specific product was expected using these primers. The thermal cycling conditions of PCR amplification were as follows: 94 °C for 2 min, 35 cycles of 94 °C for 30 s, 58 °C for 30 s and 72 °C for 30 s, and 72 °C for 5 min. The PCR products were analyzed by electrophoresis in a 2% agarose gel. Purified PCR products were subjected to two directional Sanger sequencing and BLAST search to confirm the specificity of the primers. 

The infection rate of VDV-2-China was determined by the proportion of the positive PCR results among all the tested individuals in each of the four groups: (1) *Varroa* mite, (2) *A. mellifera* and *A. cerana* adult workers, (3) *A. mellifera* worker pupae with low *Varroa* parasitism, and (4) *A. mellifera* worker pupae with high *Varroa* parasitism. For the worker pupae samples, to avoid false positive results caused by possible contamination of *Varroa* offspring, additional PCR amplifications were performed with primers targeting *Varroa tublin* gene (Forward: 5′- TCCTCGACGTTGTGCGTAAA-3′; Reverse: 5′- GCAAGGTTCCCATACCCGAA-3′). None of the honeybee worker pupae samples were found to be contaminated by *Varroa* mites. 

### 2.6. Infectivity Bioassay

Due to the ubiquitous presence of the Varroa mites in honey bee colonies, it is very possible that hive materials including food could also be contaminated by the virus and honey bees can acquire the virus via indirect food-borne horizontal transmission. Therefore, we assessed the possibility and likelihood of the VDV-2-China replication in honey bees via virus feeding.

To determine if honey bees could serve as a biological host to support the VDV-2-China replication and the infectivity of VDV-2-China in the host of the *A. mellifera* and *A. cerana*, a laboratory inoculation experiment was conducted. Approximately about 1000 adult *Varroa* mites were collected from the *A. mellifera* colonies maintained at an apiary of the College of Animal Sciences, Zhejiang University, Hangzhou, China. Collected mites were then ground into fine powder in liquid nitrogen and homogenized with 5 mL of phosphate-buffered saline (1 × PBS) in a tissue grinder. The homogenized mixture was centrifuged at 10,000 rpm at 4 °C for 40 min and the supernatant was passed through a 0.20 μm cell filter and stored at 4oC for the subsequent inoculation. 

The concentration of VDV-2-China in the purified solution described above was determined by absolute quantification using the standard curve method [73]. The purified VDV-2-China specific amplicon was incorporated into a pMD 18-T Vector (Takara Biomedical Technology, Beijing, China) following manufacturer’s protocol. The concentration of VDV-2-China was determined to be 9.91 × 10^4^ genome copies/μL.

A group of newly emerged *A. mellifera* (N = 75) and *A. cerana* (N = 75) adult workers were fed individually with 3 μL of virus solutions and transferred into rearing cages. Each cage consisted of 25 viral inoculated bees, resulting in three cages for *A. mellifera*, and three cages for *A. cerana*. Another group of newly emerged *A. mellifera* (N = 75) and *A. cerana* (N = 75) workers were fed with 3μL of PBS and used as a negative control. All the rearing cages were kept in an incubator (Stik, Shanghai, China) at a temperature of 30 ± 0.5 °C and a relative humidity of 70 ± 5%. A freshly made 50% (*w*/*v*) sucrose solution was provided for the honey bees every day. From each rearing cage, three bees were collected 12 h, two days, four days, six days, and eight days post treatment and subjected to the subsequent RNA extraction and strand-specific quantitative PCR (RT-qPCR).

In order to determine the likelihood and ability of VDV-2-China to replicate in *A. mellifera* and *A. cerana*, RNA samples from the VDV-2-China inoculated bees and negative control were analyzed for the presence and abundance of negative-stranded RNA of VDV-2 China, a replicative intermediate, using the strand-specific reverse transcription coupled with RT-qPCR. For each sample, the first strand cDNA was synthesized from total RNA with Tag- VDV-2-China forward primer (5′agcctgcgcaccgtggGCCCCATCGACTTCACGATA-3′) where the lowercase letters corresponding to Tag sequences [74]. The synthesized cDNAs were then purified using a MinElute reaction cleanup kit (Qiagen, Germany) before PCR amplification to avoid false-positive results. cDNA derived from negative-stranded viral RNA was PCR amplified with Tag primer (5′-AGCCTGCGCACCGTGG-3′) and VDV-2-China reverse primer (5′-CATGCGCACACCAATCTCAC-3) using SYBR^®^ Premix Ex TaqTM (Takara Biomedical Technology, Beijing, China) [73]. To normalize the qPCR result, amplification of a housekeeping gene, RPS5 (RPS5-F: 5′-AATTATTTGGTCGCTGGAATTG-3′; RPS5-R: 5′-TAACGTCCAGCAGAATGTGGTA-3′) was performed for each sample and a length of 115 bp of RPS5 fragment was expected using this pair of primers. The comparative Ct method (2^−ΔΔCt^) method [75] was used for calculating the fold change of VDV-2-China replication at different time points post inoculation in *A. mellifera* and *A. cerana*. One-way analysis of variance (ANOVA) was used to determine whether there is a statistical significance in the fold change of VDV-2-China replication among different time points post inoculation and Tukey’s honestly significant difference (HSD) test was used for all pairwise comparisons among different time points. A *p*-value ≤ 0.05 was considered statistically significant.

## 3. Results

### 3.1. A New Strain of Varroa destructor Virus-2 (VDV-2) Found in Varroa destructor 

**Figure 1:**
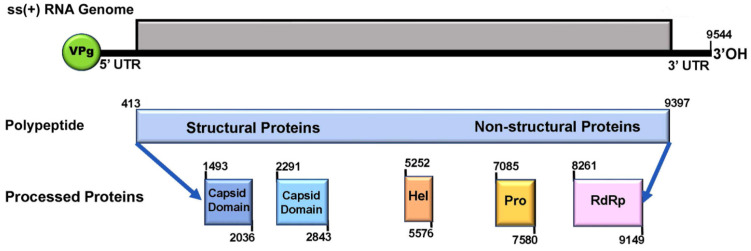
Schematic representation of the *Varroa destructor* virus-2-China (VDV-2-China) genome organization. The non-structural and structural proteins are encoded by a single open reading frame (ORF) and expressed as a polypeptide. The approximate positions of the helicase (Hel), protease (Pro), and replicase (RdRp) domains in the non-structural proteins encoded by 3′ end of ORF and the major capsid proteins domains in the structural proteins encoded by 5′ end of ORF are shown. The protein domains were defined by pairwise amino acid sequence alignment between VDV-2-China and VDV-2-UK (QFS19921) whose functional regions have been identified and annotated and further confirmed by Pfam matches.

The transcriptome assembly on the RNA reads led to a contiguous full-length viral genome. The single-stranded positive-sense RNA genome is 9544 nt long and contains a single open reading frame (ORF). The ORF encoding a polyprotein of 2995 amino acid residues is flanked by approximately 413 nt of 5′-UTR and 147 nt of 3′-UTR, which is typical for the genome organization of *Iflaviridae*. The C-terminal portion of the polyprotein possesses consensus sequences in the order of helicase, protease and RNA-dependent RNA polymerase, similar to those of *iflaviruses*. Two conserved functional motifs, rhinovirus-like (rhv) motifs belonging to the picornavirus capsid protein domain-like were identified in the N-terminal portion of the polyprotein (Figure 1). The BLAST search returned the best match to *Varroa destructor* virus 2-UK (MK795517) with an 82.96% nucleotide sequence identity (95% query coverage) and an 88.95% amino acid sequence identity (100% query coverage). The BLAST search returned a second-best match to *Varroa destructor* virus 2–Israeli (NC-04061) with a 78.58% nucleotide sequence identity (90% query coverage) and an 85.75% amino acid sequence identity (85% query coverage). Due to the revealed high level of sequence similarity with the VDV-2, we named the virus as *Varroa destructor* virus 2–China (VDV-2-China). 

### 3.2. The Phylogenetic Relationship of VDV-2-China with Other Viruses in the Order of Picornavirales and in the Family of Iflaviridae

**Figure 2:**
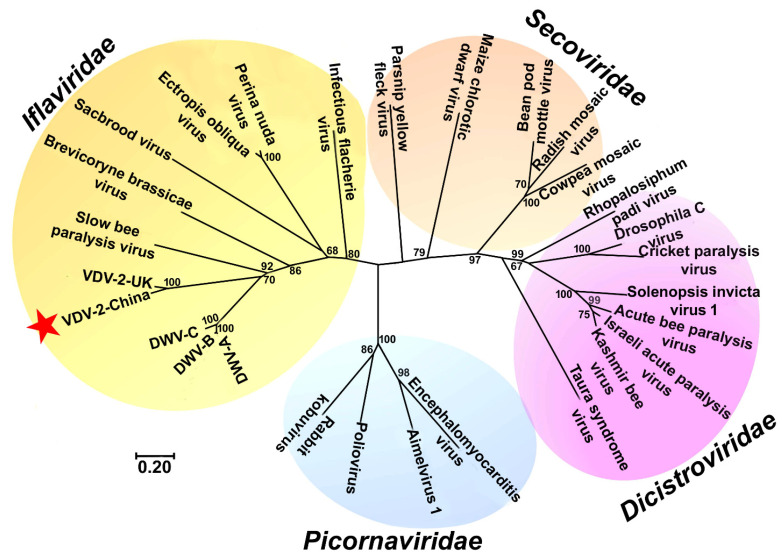
Unrooted phenogram showing the relationship of VDV-2-China with other viruses in the families *Iflaviridae*, *Dicistroviridae*, *Secoviridae*, and *Picornaviridae*. The phylogenetic analysis was conducted in MEGA 7 based on the amino acid sequences of the putative structural polyprotein using the neighbor-joining method. The tree is drawn to scale, with the same unit used for the branch lengths as those of the evolutionary distances that are used to infer the phylogenetic tree. The scale bar shows the number of substitutions per base. The neighbor-joining phenogram was bootstrapped 1000 times with values greater than 60% given at the nodes.

The phylogenetic tree constructed with the amino acid sequences of structural capsids of VDV-2-China (900 amino acids of the N-terminal region of the polyprotein) showed that VDV-2-China was distantly related to other members of the Dicistrovidae, Secoviridae, and Picornaviridae families and clustered together with other members of Iflaviridae. Of all the members of Iflaviridae, VDV-2-China is the most closely related to VDV-2-UK (Figure 2). The sequence identity of the capsid proteins between VDV-2-China and VDV-2-UK is 92.2%. However, the sequence identity of the capsid proteins between VDV-2-China and VDV-2-Israeli is below 75%.

**Figure 3:**
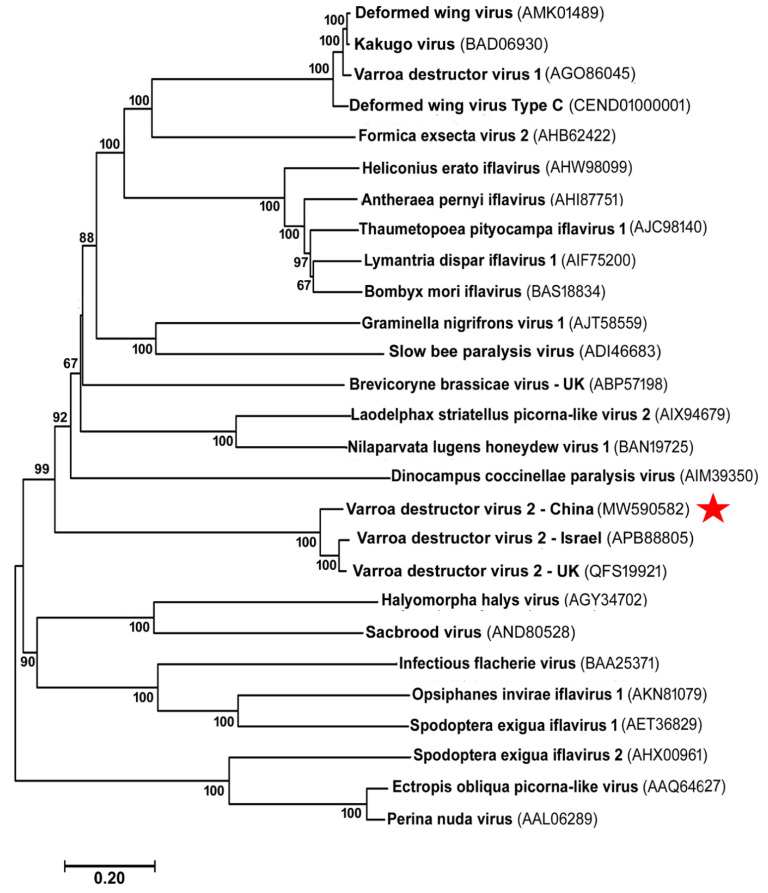
Phylogenetic tree of *iflaviruses*. The phylogenetic analysis was conducted in MEGA 7 based on the amino acid sequences of the polyprotein. The tree was constructed by the maximum likelihood method. The tree is drawn to scale, with branch lengths measured using the same units as those of the evolutionary distances that are used to infer the phylogenetic tree. The scale bar shows the number of substitutions per base. The reliability of the tree topology was determined by the bootstrap analysis (1000 replicates). The bootstrap values that were greater than 60% were given at the nodes. The names and accession numbers of the *iflavirus* taxa are shown and the red star is used to mark VDV-2 China.

The phylogenetic tree that was constructed with the amino acid sequences of RdRp at the C-terminal part of the polyprotein of all *iflaviruses* revealed that VDV-2-UK and VDV-2-Israel were clustered together into the same clade. While VDV-1, DWV, and VDV-2 are closely associated with honey bee colonies, they are not the closest relatives in the phylogenetic tree. The clade of VDV-2 strains is nested within a clade of *Graminella nigrifrons* virus 1, a virus isolated from black-faced leafhopper, Graminella nigrifrons, and SBPV infecting honey bees. Compared to DWV and VDV-1, VDV-2 strains were found to be located more closely to the root of the tree, implying that DWV and VDV-1 may be more advanced than VDV-2 in their evolution (Figure 3).

### 3.3. Prevalence and Incidence of VDV-2-China in Field-Collected Varroa and Bee Samples

**Figure 4:**
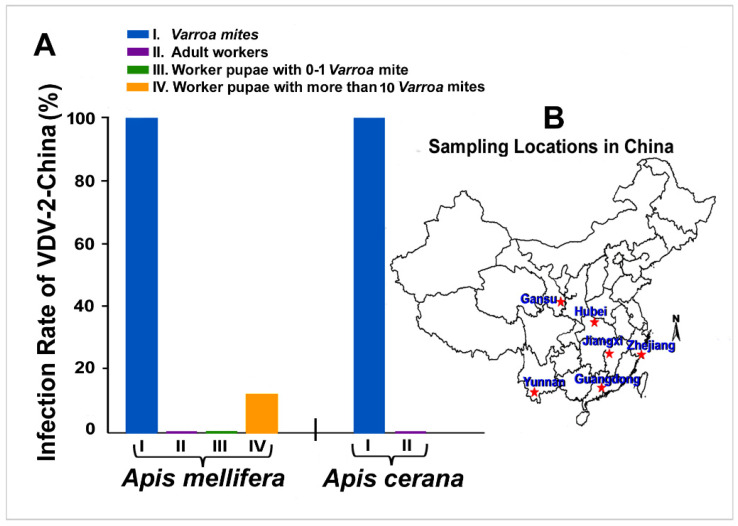
VDV-2-China infection in different regions of China under field conditions. (**A**) Infection rate of VDV-2-China in *Varroa* mites and adult workers from both *A. mellifera* and *A. cerana* colonies and worker pupae with low levels of *Varroa* parasitism, and worker pupae with high levels of *Varroa* parasitism from *A. mellifera* colonies. (**B**) A map of China showing the sampling location of the study. *A. mellifera* and *A. cerana* worker samples were collected in six provinces located in south central and eastern regions of China, including Gansu, Hubei, Jiangxi, Zhejiang, Yunnan, and Guangdong.

The examination of the field-collected *Varroa* samples showed that VDV-2-China could be detected in 100% of the *Varroa* mites collected from both *A. mellifera* colonies (N = 40) and *A. cerana* colonies (N = 28). Of all the adult workers collected from both *A. mellifera* (N = 180) and *A. cerana* colonies (N = 180) from six provinces, none of the samples was found to be positive for VDV-2-China. Of all the worker pupae with high levels of *Varroa* parasitism collected from *A. mellifera* colonies (N = 24), 12.5% of the *A. mellifera* worker pupae were found to be infected with VDV-2-China, while none of the *A. mellifera* worker pupae with low levels of *Varroa* parasitism (N = 24) tested positive for the virus (Figure 4).

### 3.4. Replication of VDV-2-China in A. mellifera and A. cerana

**Figure 5:**
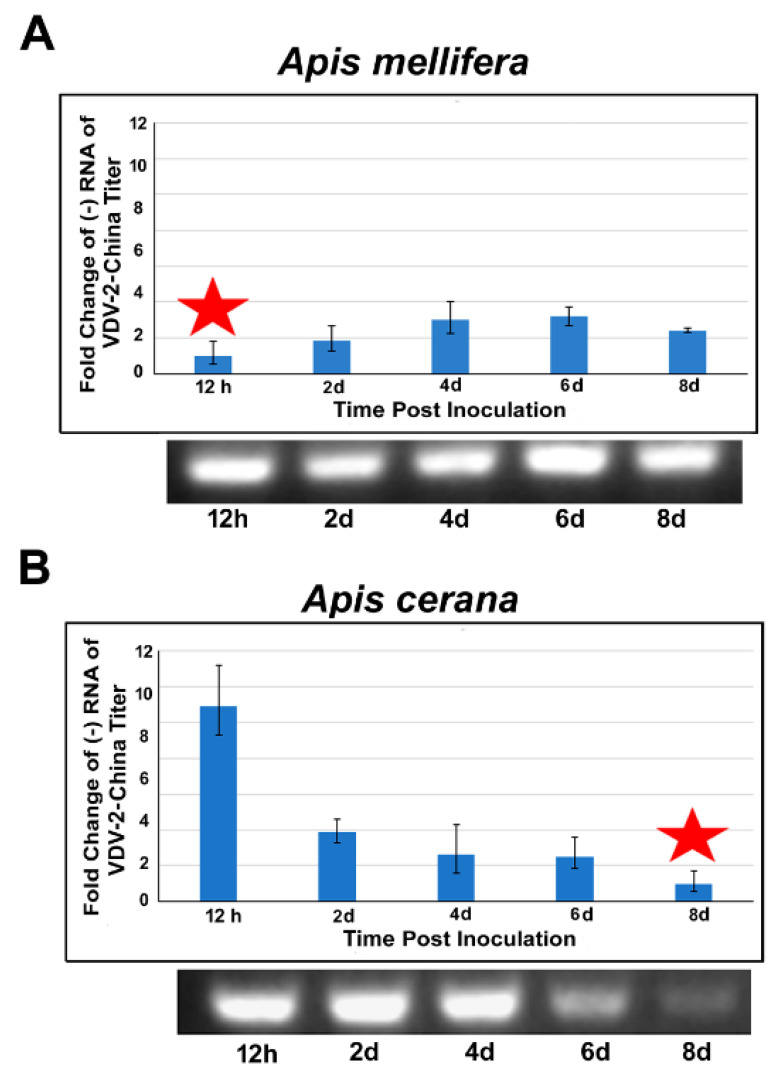
Fold change of the negative strand RNA of VDV-2-China titer in *A. mellifera* (**A**) and *A. cerana* (**B**) at different time points post inoculation. The fold change was expressed as an n-fold difference relative to the calibrator that had the lowest concentration and is equal to 1 (marked by a star) by the 2^–∆∆Ct^ method. The error bars on the barplot represent range of fold change in VDV-2-China titer (2^−ΔΔCt−S^–2^−ΔΔCt+S^) where S is the standard deviation of the ΔΔCt value. The different lowercase letters above the bars indicate the statistically significant difference (*p* ≤ 0.05, one-way analysis of variance (ANOVA) and Tukey’s honestly significant difference (HSD) test) among different time points post inoculation. A representative gel image of the PCR products of strand-specific RNA of VDV 2-China amplified by tagged RT-PCR at each time points post inoculation is shown below the bar chart for both *A. mellifera* and *A. cerana*.

While no negative-strand RNA of VDV-2-China could be detected in the negative control groups of *A. mellifera* and *A. cerana* at any time point post-inoculation, a negative-strand RNA of VDV-2-China was detected in the *A. mellifera* and *A. cerana* bee 12 h after the inoculation of VDV-2-China through oral feeding. However, VDV-2-China displayed different replication dynamics between the two bee species. In *A. mellifera*, an increase in the measured negative-strand RNA of VDV-2-China was observed on day 2 post-infection and this trend continued until day 6 post-inoculation. The titer of the negative-strand RNA of VDV-2-China on day 6 was 3.4-fold higher than at 12 h post-inoculation. The titer of the negative-strand RNA of VDV-2-China began declining after day 6 post-infection (Figure 5A). In contrast, the mean negative-strand RNA of VDV 2-China in *A. cerana* declined in a time-dependent manner from 12 h post-infection. The titer of the negative-strand RNA of VDV-2-China on day 2 post-infection was found to have decreased by half, and it decreased more than nine-fold on day 8, compared to the titer of the negative strand of the viral RNA 12 h post-infection (Figure 5B).

## 4. Discussion

Of the various disease agents threatening honey bee health and well-being, RNA viruses that have high mutation rates and undergo rapid evolution and natural selection have often been implicated in colony losses and they are of particular concern to beekeepers worldwide. The development of next-generation sequencing technologies that are culture-independent and capable of providing massive amount of parallel sequencing has revolutionized our ability to discover new viruses and elucidate evolutionary relationships between viruses. Using Illumina HiSeq sequencing technology, we conducted metagenomic studies of microbial communities that are associated with parasitic *Varroa* mites, which led to the identification of a new strain of VDV-2 [56,57] (VDV-2-China). The complete genome sequence analysis of VDV-2-China revealed characteristic features that are unique to the Iflaviridae family. Based on NCBI BLAST searches and phylogenetic analyses, VDV-2-China is most closely related to VDV-2-UK among all of the members of the Iflaviridae family. According to the International Committee on Taxonomy of Viruses (ICTV), species of the Iflaviridae family are demarcated by host range and a <90% amino acid identity in the sequence of the capsid protein precursor [76]. In light of this over 90% sequence homology was displayed between VDV-2-China and VDV-2-UK with regard to the capsid precursor, therefore, we conclude that VDV-2-China is a new strain of VDV-2.

The *Varroa* mite has a long history of association with honey bee hosts. It was first reported in relation to its natural host, *A. cerana*, in 1904 and was believed to have made the jump from *A. cerana* to *A. mellifera* at least twice, probably around the 1950s [8,77]. While a great deal of attention has been paid to the devastating damage that *Varroa* has inflicted on honey bees and honey bee viruses that were vectored and activated by the *Varroa* mite, limited attention has been paid to the viruses that are harbored by the mites and are mostly absent from honey bees as well as the effects of these viruses on their parasite hosts until recent years [56,57,58]. In 2016, an Israeli strain of VDV-2 was first reported and was later found in North Africa and Thailand [58]. It was described that the Israeli strain of VDV-2 was exclusively associated with *Varroa* and was absent in *A. mellifera* [57]. Meanwhile, a more recent study discovered a UK strain of VDV-2 that was also found to be prevalent in Spain and provided evidence that the UK strain of VDV-2 was able to replicate in the mite, yet not in the bee, suggesting that the virus selectively infects the *Varroa* mite [56]. Generally, our results are consistent with previous reports and showed that VDV-2-China was constantly present in *Varroa* populations. This was demonstrated when VDV-2 was detected in all of the *Varroa* mites collected from both *A. mellifera* and *A. cerana* colonies. All of the *A. mellifera* and *A. cerana* adult workers that were distributed in six provinces in China as well as *A. mellifera* worker pupae exposed to a low level of *Varroa* mites tested negative for VDV-2-China, clearly indicating that the strain is predominantly a Varroa-adapted virus. However, in our study, VDV-2-China was detected in 12.5% of the *A. mellifera* worker pupae that were parasitized by more than 10 *Varroa* mites, bringing into play the possibility of a new scenario where VDV-2 could be transmitted to the honey bees during heavy *Varroa* infestations. Further research is warranted to investigate the capacity of the *Varroa* mite as a vector for transmitting VDV-2-China to honey bees. 

The infectious disease process includes three key components: (1) an infectious agent in its natural reservoir, (2) the acquisition of the disease agent by susceptible hosts via transmission, and (3) the efficient replication of a virus in the infected host. Infectivity and pathogenesis of a pathogen can be described as the ability of the pathogen to evade a host’s immune responses and to use the host’s resources to replicate and establish a persistent infection. Like any other positive-stranded RNA virus, the replication of VDV-2-China takes place through the production of negative-stranded intermediate; therefore, the presence of a negative-stranded RNA is proof of active viral replication [78]. Our results clearly showed that VDV-2-China could replicate in both *A. mellifera* and *A. cerana* under controlled laboratory conditions. Nevertheless, the observed differences in terms of the replication dynamics of VDV-2-China between *A. mellifera* and *A. cerana* reflect the respective hosts’ variation with regard to their susceptibility to the virus replicating. The latter species is believed to have some natural defense mechanisms against *Varroa* mite infestation due to a relatively longer history of host–parasite co-evolution [79]. While the titer of the negative-stranded RNA of VDV-2-China increased in *A. mellifera* continuously within six days after the initial inoculation, the concentration of the negative-stranded intermediate of the virus decreased by half on day two and more than nine-fold on day eight post-infection in *A. cerana*. This result, together with the observation that VDV-2-China was not detected in any of the examined adult workers of *A. cerana*, suggests that *A. cerana* is not a permissive host for VDV-2-China and it may possess some defense mechanisms against the virus’s replication. On the other hand, the demonstration of VDV-2-China replication in *A. mellifera* implies that *A. mellifera* could be a biological host for the virus and that the *Varroa* mite could possibly facilitate the transmission of the virus directly into honey bees, posing a potential threat to the health of *A. mellifera*.

In sum, our present study reports the discovery of a new strain of VDV-2 and supports previous findings that VDV-2 predominantly infects *Varroa* mites. We also provide evidence that *A. mellifera* is more susceptible to the VDV-2-China infection than *A. cerana*. However, certain questions still remain: is VDV-2-China harmful to its parasite host? While VDV-2-China could replicate in *A. mellifera*, what is the infective dose of VDV-2-China that will cause disease in honey bees? Future investigations to define the virus–host interactions that regulate the replication and pathogenesis of VDV-2-China in the host would yield critical insights into the evolutionary change of the virus’ prevalence, distribution, and virulence, thereby contributing to an effective disease management program for honey bees. 

## Data Availability

The viral genome sequence of VDV-2-China was submitted to Genbank with an accession number (MW590582) and the raw RNA sequencing data of *Varroa destructor* from *A. mellifera* were submitted into the NCBI’s Sequence Read Archive (SRA) database with ID # SUB9162165.
